# Flagellin-Induced Immune Response in Human-Induced Pluripotent Stem Cell-Derived Cardiomyocytes

**DOI:** 10.3390/ijms241813933

**Published:** 2023-09-11

**Authors:** Goekhan Yuecel, Xiaobo Zhou, Linda Terkatz, Angela Wendel, Julius Reinhardt, Ibrahim El-Battrawy, Katherine Sattler, Lukas Cyganek, Jochen Utikal, Harald Langer, Ruediger Scharf, Daniel Duerschmied, Ibrahim Akin

**Affiliations:** 1 Department of Cardiology, Angiology, Haemostaseology and Medical Intensive Care, University Medical Center Mannheim, Medical Faculty Mannheim, Heidelberg University, 68167 Mannheim, Germany; 2 European Center for AngioScience (ECAS) and German Center for Cardiovascular Research (DZHK) Partner Site Heidelberg/Mannheim, 68167 Mannheim, Germany; 3 Department of Pediatric Surgery and Pediatric Urology, Children’s Hospital of Cologne, 50735 Cologne, Germany; 4 Department of Cardiology and Angiology, Bergmannsheil University Hospitals, Ruhr University of Bochum, 44791 Bochum, Germany; 5 DZHK (German Center for Cardiovascular Research), Partner Site, 37075 Göttingen, Germany; 6 Stem Cell Unit, Clinic for Cardiology and Pneumology, University Medical Center Göttingen, 37075 Göttingen, Germany; 7 Skin Cancer Unit, German Cancer Research Center (DKFZ), 69120 Heidelberg, Germany; 8 Department of Dermatology, Venereology and Allergology and DKFZ Hector Cancer Institute, University Medical Center Mannheim, University of Heidelberg, 68167 Mannheim, Germany

**Keywords:** hiPSC-CM, PAMP, TLR, innate immunity, septic cardiomyopathy

## Abstract

Pathogen-associated molecular patterns (PAMPs) are involved in the pathogenesis of septic cardiomyopathy through a toll-like receptor (TLR)-mediated immune response. Human-induced pluripotent stem cell-derived cardiomyocytes (hiPSC-CMs) can reflect the innate immune abilities of cardiomyocytes. Therefore, hiPSC-CMs may provide an attractive tool with which to study PAMP-induced alterations in cardiomyocytes. HiPSC-CMs from two different healthy donors were exposed to the PAMP flagellin (FLA) at different doses and exposure times. Alterations in the expression levels of distinct inflammation-associated cytokines, intracellular inflammation pathways including TLR5 downstream signaling, reactive oxygen species levels and surface antigen composition were assessed using PCR, ELISA and FACS techniques. Higher doses of flagellin increased the expression levels of inflammation-associated cytokines like TNFα (*p* < 0.01) and downstream signaling molecules like caspase-8 (*p* < 0.05). TLR5 expression (*p* < 0.01) and TLR5 fluorescence proportion (*p* < 0.05) increased in hiPSC-CMs after prolonged FLA exposure. FLA-induced innate immune response processes in cardiomyocytes might be detectable with an hiPSC-CMs-based in vitro model.

## 1. Introduction

Sepsis remains a serious life-threatening organ dysfunction caused by a dysregulated host response to infection [1]. Bacterial sepsis is most frequently induced by either Gram-negative or -positive bacteria, which can appear in the bloodstream during septic endotoxinemia [2]. In addition to affecting septic circumstances, bacteria can directly affect the heart and lead to bacterial myocarditis, which is less common than the viral form. However, several bacteria, such as Salmonella or Borrelia, are able to cause bacterial myocarditis [3]. PAMPs are essential parts of those bacteria and key molecules for microbial pathogenicity. They can activate human immune responses in different cellular and humoral pathways [4]. PAMP recognition is mediated through pattern recognition receptors (PRRs) expressed by several cell types, including antigen-presenting cells (APCs). PRRs play a crucial role in the consecutive immune responses. Particularly, TLRs, as members of an important membrane-bound subfamily of PRRs, are involved in the coordination of innate and adaptive immune responses to PAMP appearance [5,6]. Several PAMPs, like lipopolysaccharides (LPS), lipoteichoic acid (LTA) or FLA, are known to induce various cytokine responses mediated by corresponding TLRs and following pathways. In addition to TLR-dependent extracellular recognition, intracellular recognition of endocytosed PAMP can furthermore lead to inflammasome activation and additional immune responses [7].

Septic spread can potentially affect every organ. Sepsis-induced cardiac inflammatory alterations are summarized within sepsis-induced cardiomyopathy (SCM), while alterations from bacterial infectious myocarditis without further septic conditions are described as inflammatory cardiomyopathy [3]. Without any commonly accepted definition, SCM is most often described as echocardiographic reversible acute biventricular systolic and/or diastolic dysfunction and reduced contractility. Several further parameters are known to be relevant for SCM [8]. While SCM was suspected to be a hemodynamic complication of sepsis for a long time, proof of TLR4 in human cardiomyocytes led to the suspicion that cardiomyocytes might be directly involved in endotoxinemia-related immune responses [9]. Even an overwhelming local inflammatory response due to those APC-like attributes in cardiomyocytes was discussed, addressing a potential cardiac pathophysiological correlate of general dysregulated host response in sepsis [10,11]. On the other hand, patients with bacterial myocarditis and inflammatory cardiomyopathy can present with similar clinical findings [12]. However, PAMP-based in vitro modeling is commonly used to enlighten local host response pathways in various tissues [13,14]. Concerning cardiomyocytes and heart tissue, several TLRs may have already been revealed to be expressed in cardiomyocytes [15], addressing a broad range of distinct PAMP-like LPS, LTA or FLA [16,17,18,19,20]. In this context, hiPSCs and hiPSC-CMs provide a newer option with which to explore cardiomyocyte signaling pathways, and hiPSC-CM-based modeling of cardiomyopathies is commonly established [21,22]. Hence, this study aimed to reveal for the first time whether FLA would be able to induce inflammatory responses in hiPSC-CMs.

## 2. Results

### 2.1. Generation of HiPSC-CM

**Figure 1:**
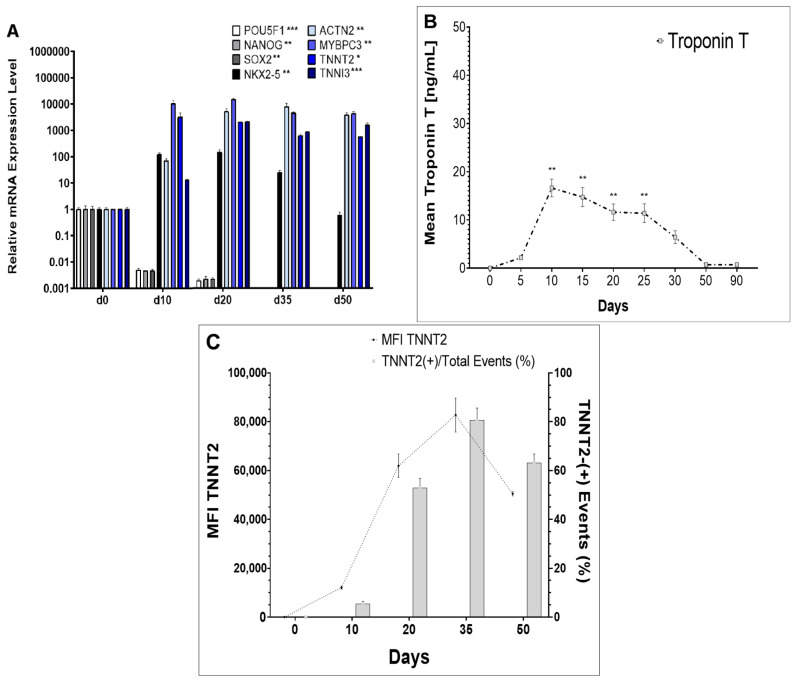
(**A**) PCR analysis during cardiac differentiation process from day 0 to day 50. Values are given as mean fold change +/− SD compared to hiPSC status on d0. * = *p* < 0.05, ** = *p* < 0.01, *** = *p* < 0.001 (one-way ANOVA). POU5F1 = Oct3/4 = POU class 5 homeobox 1, NANOG = Nanog homeobox, SOX2 = SRY-box transcription factor 2, NKX2-5 = NK2 homeobox 5, ACTN2 = alpha actinin 2, MYBPC3 = myosin-binding protein C3, TNNT2 = troponin T cardiac type, TNNI3 = troponin I cardiac type. Ct values for NANOG, SOX2 and POU5F1 on d20, d35 and d50 were >35. (**B**) ELISA analysis during cardiac differentiation process from day 0 to day 90. Troponin T (TnnT) levels (mg/mL) in hiPSC supernatants were measured and compared to baseline hiPSC status on day 0. Values are given as mean fold change +/− SD, ** = *p* < 0.01 (Dunnett’s multiple comparisons test vs. day 0). (**C**) Illustration of mean fluorescence intensity (MFI) signal for AF647-conjugated anti-troponin T (TNNT2, cardiac type) antibody during cardiac differentiation process from day 0 to day 50 (line). The right-side panel shows the corresponding ratio of TNNT2-positive events related to all events (grey bars). The highest levels for MFI and TNNT2-positive events were detectable on day 35.

Before starting inflammatory studies, we confirmed the presence of hiPSC-CMs in our differentiation protocol. The generation of hiPSC-CMs was described by PCR analysis for 50 days after the start of the differentiation process. We showed decreasing expression levels for the pluripotency-associated genes POU5F1 (POU class 5 homeobox 1), NANOG (Nanog homeobox) and SOX2 (SRY-box transcription factor 2) compared to hiPSC status; gene expression was no longer detectable 20 days after the start of the differentiation process (*p* < 0.05 each, one-way ANOVA, Figure 1A). The expression level of the cardiomyocyte- and cardiomyogenesis-associated genes TNNT2, ACTN2 (alpha actinin 2), NKX2-5 (NK2 homeobox 5), MYBPC3 (myosin-binding protein C3) and TNNI3 (cardiac muscle troponin I) significantly increased after the start of the cardiac differentiation process (*p* < 0.05 each, one-way ANOVA, Figure 1A). We further detected significantly elevated extracellular TNNT2 with ELISA measurements of supernatants during the cardiac differentiation process (*p* < 0.05, one-way ANOVA, Figure 1B). Corresponding intracellular TNNT2 was assessed with FACS measurements, showing the highest level of TNNT2-positive events around d20–35 (Figure 1C) and striated morphology due to sarcomere formation in immunostaining (Supplementary Figures S2–S5). Cell beating was visible through daily microscope evaluation 7–10 days after the start of the differentiation process.

### 2.2. Inflammation-Associated Gene Expression

**Figure 2:**
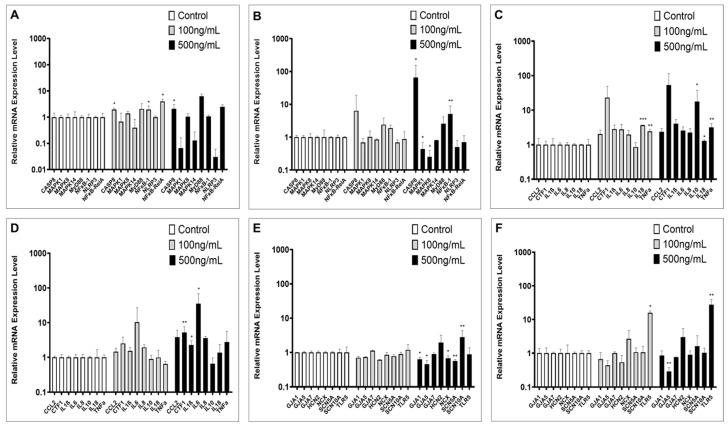
PCR analysis of distinct gene groups in hiPSC-CMs after FLA exposure with either 100 ng/mL or 500 ng/mL versus control for 6 h (**A**,**C**,**E**) and 48 h (**B**,**D**,**F**). Values are given as mean fold change +/− SD. * = *p* < 0.05, ** = *p* < 0.01, *** = *p* < 0.001 (Dunnett’s multiple comparisons vs. negative control). Intracellular inflammation-signaling-associated genes: CASP8 = caspase-8, MAPK-1/-8/-14 = mitogen-activated protein kinase 1/8/14, MyD88 = MYD88 innate immune signal transduction adaptor, NF-κB-1/-RelA = nuclear factor kappa B subunit 1/subunit RelA, NLRP3 = NLR family pyrin domain containing 3. Cytokines: CCL2 = C-C motif chemokine ligand 2, CTF1 = cardiotrophin 1, IL1β/6/8/10/18 = interleukin 1β/6/8/10/18, TNFα = tumor necrosis factor α. Receptor and ion-channel genes: GJA1/5 = gap junction protein alpha 1/5, GJA7 = gap junction protein gamma 1, HCN2 = hyperpolarization-activated cyclic nucleotide-gated potassium and sodium channel 2, NCX = solute carrier family 8 member A1, SCN5A/10A = sodium voltage-gated channel alpha subunit 5/10, TLR5 = toll-like receptor 5. Calcium voltage-gated channel subunit alpha 1 C (CACNA1C), hyperpolarization-activated cyclic nucleotide-gated potassium and sodium channel 4 (HCN4). IL12, HCN4, SCN3B, potassium voltage-gated channel subfamily D member 3 (KCND3) and potassium inwardly-rectifying channel subfamily J member 3 (KCNJ3) are not listed due to missing significant alterations.

Expression levels of key genes from innate inflammatory signal transduction were measured in hiPSC-CMs cell culture after exposure to FLA at either 100 ng/mL or 500 ng/mL. With regard to short-term incubation (6 h), caspase-8 (CASP8) and nuclear factor kappa B subunit 1/subunit RelA (NF-κB-1 and -RelA) showed significant alterations for 100 ng/mL, while only CASP8 was significantly increased with both doses (*p* < 0.05, Dunnett’s multiple comparisons, Figure 2A). Long-term exposure to FLA (48 h) showed increasing expression levels, with 500 ng/mL for CASP8 and NF-κB-1 only, but decreasing expression for mitogen-activated protein kinase 1/8 (MAPK1 and MAPK8) (*p* < 0.05, Dunnett’s multiple comparisons, Figure 2B). Cytokine gene expression was assessed with the same values and exposure times. Six hours of exposure revealed a dosage-independent increase for IL18 and TNFα, while the increased expression of IL10 was only detectable with FLA 500 ng/mL (*p* < 0.05, Dunnett’s multiple comparisons, Figure 2C). Prolonged exposure revealed increasing expression levels for IL1β, IL6 and cardiotrophin 1 (CTF1) with 500 ng/mL (*p* < 0.05, Dunnett’s multiple comparisons, Figure 2D). CCL2 and IL8 showed no relevant alterations. Major ion channels of cardiac electrophysiology were further checked for FLA-induced gene expression alterations. Short-term exposure showed decreases for gap junction antigen (GJA) 1, GJA5, sodium–calcium exchanger (SLC8A1 = CX) and sodium voltage-gated channel alpha subunit 5 (SCN5A) with 500 ng/mL FLA, while a significant increase was only detectable for SCN10A. Under long-term exposure, there was only an increase for TLR5 detectable (*p* < 0.01, Dunnett’s multiple comparisons, Figure 2E,F).

### 2.3. Supernatant Composition

**Figure 3:**
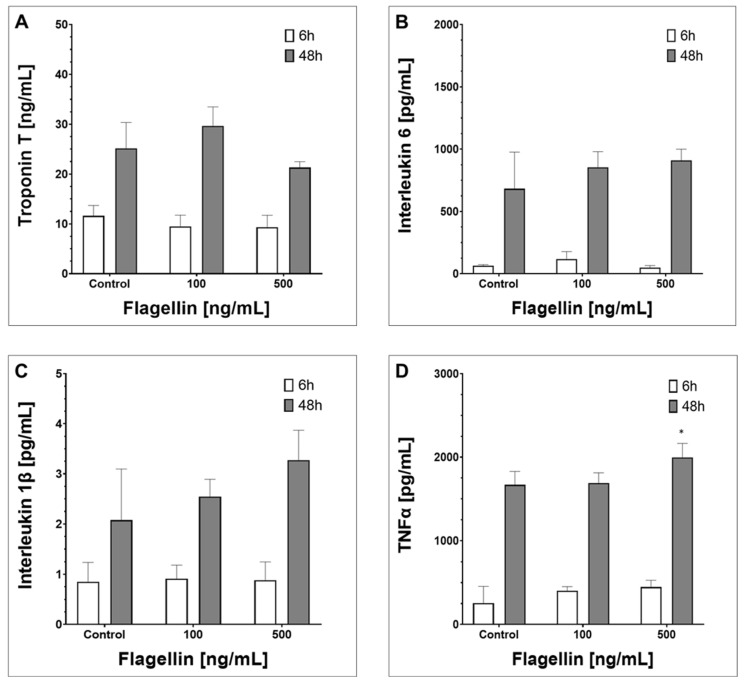
ELISA analysis of hiPSC-CMs supernatants after 48 h incubation with either negative control (RPMI w/o FLA), or 100 ng/mL or 500 ng/mL FLA. (**A**) Troponin T (ng/mL). (**B**) Interleukin 6 (pg/mL). (**C**) Interleukin 1β (pg/mL). (**D**) TNF α (tumor necrosis factor α, pg/mL). Values are given as mean concentration +/− SD. FLA and time influence were checked separately (two-way ANOVA, factors time and FLA concentration). * = *p* < 0.05. *p*-values: 3A—Troponin T: FLA = 0.18, time = 0.001, interaction = 0.19. 3B—Interleukin 6: FLA = 0.34, time = 0.016, interaction = 0.64. 3C—Interleukin 1β: FLA = 0.11, time = 0.022, interaction = 0.54. 3D—Tumor necrosis factor α: FLA = 0.34, time = 0.01, interaction = 0.1.

We evaluated extracellular protein concentrations using ELISA in hiPSC-CMs supernatants undergoing FLA exposure for 6 h and 48 h, respectively. FLA did not influence troponin T concentration, whereas the factor exposure time significantly increased extracellular detectable troponin T (two-way ANOVA factors FLA/time, *p* = 0.18 vs. *p* < 0.01, interaction: 0.19, Figure 3A). Similar results were detectable for IL1β and IL6, showing significant alterations in protein concentration for the factor time only, but not for the factor FLA (Figure 3B,C), which was only found to be relevant for TNFα (two-way ANOVA factors FLA/time, *p* = 0.03 vs. *p* < 0.01, interaction *p* = 0.1, Figure 3D).

### 2.4. Surface and Intracellular Protein Levels and ROS Induction

**Figure 4:**
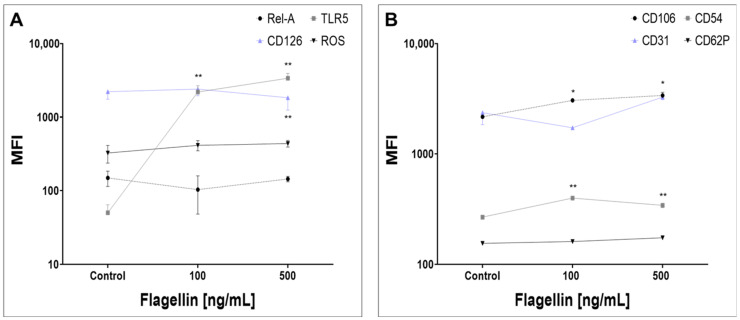
FACS measurement in hiPSC-CMs after 48 h FLA exposure with either 100 ng/mL or 500 ng/mL compared to negative control. (**A**) FLA-associated inflammatory receptors and proteins, (**B**) Inflammation-associated surface cell adhesion molecules. Values are given as mean of MFI (mean fluorescence intensity) +/−SD. Listed MFI values reflect values from single-cell cardiac troponin positive events gate. * = *p* < 0.05, ** = *p* < 0.01 (Dunnett’s multiple comparisons vs. negative control). CD = cluster of differentiation. RelA = nuclear factor kappa B subunit RelA, TLR5 = toll-like receptor 5, ROS = reactive oxygen species.

We evaluated certain surface antigen alterations in TNNT2-positive events after FLA exposure for 48 h (compare gating hierarchy, Supplementary Figure S2). We showed a dose-independent increase for TLR5 MFI (*p* < 0.01, Dunnett’s multiple comparisons, Figure 4A), whereas NF-κB-RelA and reactive oxygen species (ROS) were not influenced by FLA. CD126 decreased under 500 ng/mL FLA (*p* < 0.01, Dunnett’s multiple comparisons, Figure 4A). We further measured alterations for certain cell adhesion molecules and showed a dose-independent increase for vascular cell adhesion protein 1 (VCAM-1=CD106) and intercellular adhesion molecule 1 (ICAM-1=CD54) after FLA exposure (*p* < 0.05 for CD106 and *p* < 0.01 for CD54 vs. control, Dunnett’s multiple comparisons, Figure 4B). Platelet endothelial cell adhesion molecule 1 (PECAM-1=CD31) and platelet selectin (CD62P) were not influenced by FLA. Apoptosis/necrosis measurement and NLR family pyrin domain containing 3 (NLRP3) did not show significant alterations in FACS measurements and were not further listed due to overview reasons.

## 3. Material and Methods

### 3.1. Ethics Statement

The study was approved by the Ethics Committee of the Medical Faculty Mannheim, Heidelberg University (approval numbers: 2009-350N-MA, 2018-637N-MA) and the University Medical Center Göttingen (approval number: 10/9/15), being in accordance with the Helsinki Declaration of 1975, as revised in 1983.

### 3.2. Cell Culture with hiPSC and hiPSC-CMs

The hiPSC cell lines were generated from primary human fibroblasts derived from skin biopsies from two different healthy donors (cell lines D1 and D2). The hiPSC line D1 was generated by lentiviral particles carrying the transactivator tTA and an inducible polycistronic cassette containing the reprogramming factors OCT4 (octamer-binding transcription factor 4), SOX2 (sex-determining region Y—box 2), KLF4 (Kruppel-like factor 4) and c-MYC (MYC proto-oncogene), as previously described [23,24]. The hiPSC line D2 was generated by the use of an integration-free reprogramming method [25]. Generated hiPSCs were cultured under feeder-free conditions and fed with TeSR-E8 (Stemcell Technologies, Cologne, Germany) cell culture medium. Culture dishes and wells were coated with Matrigel (Corning, NY, USA). To investigate pluripotency status and decreasing pluripotency during differentiation, hiPSCs were subjected to a teratoma formation assay and the expression of pluripotency genes such as POU5F1 (POU class 5 homeobox 1), NANOG (Nanog homeobox) and SOX2 (SRY-box transcription factor 2) were assessed. The differentiation process into hiPSC-CMs was carried out as previously described [26]. In short, the cardiac differentiation process was started with mesoderm induction using the transcription factors CHIR99021 (Stemgent, Glasgow, Scotland), BMP-4 (R&D Systems, Minneapolis, MN, USA), Activin A (R&D Systems) and FGF-2 (MiltenyiBiotec, Bergisch Gladbach, Germany). According to our differentiation protocol, a Wnt signaling pathway inhibition was induced with the potent inhibitor IWP-4 (Stemgent). Finally, a metabolic selection based on different glucose and lactate metabolism in cardiomyocytes and non-cardiomyocytes [27] was performed by using glucose-free lactate-containing RPMI medium (Biological Industries, Kibbutz Beit-Haemek, Israel). The differentiation process was finished after 15–18 days. Afterwards, acquired hiPSC-CM were fed with RPMI 1640 Glutamax (Life Technologies, Waltham, MA, USA) containing sodium pyruvate, penicillin/streptomycin, B27 supplement (Life Technologies) and ascorbic acid (Sigma Aldrich, Taufkirchen, Germany). After a resting time of 5–7 days, cells were used for the studies around d25–30. Cell culture materials are listed in Supplementary Table S1. A schematic overview for hIPSC-CMs-based inflammatory studies is shown in Supplementary Figure S1.

### 3.3. Inflammatory Studies

The inflammatory studies were carried out at d30, after the start of differentiation. B27 supplementation was paused due to glucocorticoid content 5 days in advance to avoid potential immune-suppressive effects (after d25). FLA from *Salmonella typhimurium* was used for PAMP studies (Sigma Aldrich). The stock solutions were generated based on the manufacturer’s instructions. Following internal experiences, 6 and 48 h were used as short-term and long-term exposure times. To avoid wearing-off effects, the PAMP solution was changed after 24 h in periods of 48 h exposure duration. In cases of supernatant measurements after 48 h, changing of PAMP solution was not performed. PAMP concentration range was used following the manufacturer’s instructions and previously described values. However, to avoid subliminal effects and reveal potential effects above the threshold, respectively, a broad range of FLA concentrations were previously tested (0–2000 ng/mL), resulting in 100 ng/mL and 500 ng/mL vs. negative control for each study. Every experiment was carried out with four biological replicates (two replicates from each donor cell line); furthermore, each biological replicate was measured in duplicate (two technical replicates).

### 3.4. Polymerase Chain Reaction

The quantitative polymerase chain reaction (PCR) assays were performed as described before [22]. In short, total RNA, including DNAse, treatment was prepared using the RNeasy mini kit (Qiagen, Hilden, Germany). The RNA was reverse-transcribed to cDNA with oligo (dT)15 primers using an avian myeloblastosis virus reverse transcriptase according to standard protocols (Roche Applied Science, Penzberg, Germany). The cDNA was amplified by PCR on a Stratagene MX 3005 P real time cycler (Stratagene, Santa Clara, CA, USA) using a PCR mix with hot start Taq DNA polymerase and SYBR-Green (SibirRox Hot Mastermix, Bioron, Römerberg, Germany) in the presence of sense and antisense primers (400 nM each). The relative quantification of mRNA expression was calculated using the ΔΔCT method [28], while glycerinaldehyd-3-phosphat-dehydrogenase (GAPDH) was used as a housekeeping gene. Ct values >35 were defined as non-expression. The results are given as mean relative mRNA expression level. Material used for PCR is listed in Supplementary Table S1, while the measured primers (Qiagen, Hilden, Germany) are shown in Supplementary Table S2.

### 3.5. ELISA

Concentrations of human interleukin 1β (IL1β), interleukin 6 (IL6), tumor necrosis factor α (TNFα) and troponin T (TNNT2) were measured in the cell culture supernatants by ELISA (RayBiotech, Peachtree Corners, GA, USA) according to the manufacturer’s instructions and suggested dilution (1:2). ELISA measurements were performed with Infinite^®^ 200 PRO and Magellan™ software Version Standard 3.23 (Tecan). The used ELISA sets are listed in Supplementary Table S1. 

### 3.6. Immunohistochemistry and Fluorescence-Activated Cell Sorting

Immunohistochemistry and fluorescence-activated cell sorting (FACS) were performed as previously described [22,29]. In short, the initial steps were similar for both methods. The hiPSC-CMs were dissociated from 24-well cell culture plates. The cells were incubated with 300 μL (150 U) collagenase CLS I (Worthington, Lakewood, NJ, USA) for 40 min at 37 °C, following phosphate-buffered saline (PBS) washing (Gibco, Waltham, MA, USA) and 0.05% Trypsin-EDTA (Life Technologies) incubation for 4 min at 37 °C. Afterwards, the cells were centrifuged at room temperature with 250× *g* for 2 min in RPMI medium containing 10% fetal calf serum (FCS). The supernatant was discarded and the cells were resuspended with basic culture medium. In terms of immunofluorescence staining, the separated cells were resuspended with serum-free medium and transferred to 4-chamber glass slides (Corning, NY, USA). Then, 4% paraformaldehyde (Carl Roth, Karlsruhe, Germany) was used to fix the cells. Permeabilization was performed with 0.5% Triton X-100 (Sigma Aldrich) for 10 min and PBS containing 1% bovine serum albumin (Sigma Aldrich). After each step, the cells were washed with PBS. Finally, cell culture was incubated with several antibodies according to the manufacturer’s instructions at 4 °C in the dark. After one hour, antibodies were washed out with PBS. Nuclear staining was performed with Vectashield mounting medium with DAPI (Vector Laboratories, Newark, CA, USA). Pictures were taken with a Leica DMRE fluorescence microscope, DFC3000G/DFC 450c camera equipped with Leica Application suite 4.4 software.

For FACS analysis, the initial steps were similar. After PBS resuspension from collagenase treatment, fixation was performed with 1% formaldehyde solution (Merck, Darmstadt, Germany) and the cells were permeabilized with perm/wash buffer for 10 minutes under 1:10 dilution (BD Bioscienes, Heidelberg, Germany). Afterwards, cells were incubated with different antibodies for 30 minutes at 4 °C in the dark. After washing out the antibodies, samples were immediately measured at the CANTO II (BD Biosciences). Compensation measurement was considered, if necessary, by using OneComp eBeads (eBioscience, Waltham, MA, USA). In cases of two antibodies, the fluorescence derivative combination was always chosen with the most different absorption maxima as possible to avoid overlapping signals. All antibodies were checked for correct concentration by previous testing with titration measurements (1:10,000 to 1:10 dilution) and comparison to the negative control, respectively, by using isotype controls. FSC, SSC and further scatter-gating were applied equally for each experiment to avoid measurement bias. In general, the FACS gating hierarchy was based on total events (FSC-A and SSC-A gate), single-cell-events (FSC-A and FSC-W gate) and mean fluorescence intensity (MFI) gates depending on the antibodies used. To measure isolated signals on hiPSC-CMs events, a gating hierarchy consisting of single-cell and cardiac-troponin-positive events gating was applied (compare Supplementary Figure S2). Furthermore, reactive oxygen species (ROS) measurements were applied with a FACS assay kit (ThermoFisher, Waltham, MA, USA) and apoptosis/necrosis measurements were performed with an Annexin-V/Propiumiodid kit (BD Biosciences). A list of all antibodies used for FACS and immunofluorescence staining is attached (Supplementary Table S3). In cases of two different antibodies and distinct recommendations for incubation temperatures for each antibody, measurements were performed with both temperatures (results were listed as the mean of both measurements).

### 3.7. Statistical Analysis

If not otherwise indicated, data are shown as the mean ± SD and were analyzed using InStat 3.0© (GraphPad, San Diego, CA, USA) and SigmaPlot 11.0 (Systat, Germany). Each experiment was performed with n = 2 for each cell line; as two different healthy cell lines were used, each experiment was based on n = 4 biological replications. Furthermore, each sample was measured in two technical replications. The presented results reflect mean values from each experiment, considering biological and technical replications. By analyzing the data with the Kolmogorov–Smirnov test, it was decided whether parametric or non-parametric tests were used for analysis. For parametric data, one-way ANOVA with Dunnett’s post-test for multiple comparisons (all treated groups versus control) or testing of trends was performed. For non-parametric data, the Kruskal–Wallis test with Dunn’s multiple comparisons post-test was used. For repeated measurements, parametric one-way repeated measures ANOVA with Dunnett’s multiple comparisons post-test was applied. Comparisons of two independent groups with normal distribution were performed with the *t*-test. *p*-values <0.05 were considered significant and *p* < 0.10 (two-tailed) was considered significant by tendency, respectively. Further illustrations were performed with Microsoft Office Professional Plus Excel and PowerPoint 2016^©^ and Prism 8.0© (GraphPad, USA).

## 4. Discussion

Sepsis-induced cardiomyopathy and myocarditis with inflammatory cardiomyopathy might be life-threatening sequelae of different underlying conditions. Several pathogen-associated molecular patterns like those of bacterial flagellin play a crucial role in underlying inflammatory processes. To the best of our knowledge, this was the first study on whether FLA would be able to effect inflammatory alterations in hiPSC-CMs. We detected some inflammatory responses in hiPSC-CMs induced by FLA at various exposure times and concentrations, reflected through the gene upregulation of cytokines, intracellular TLR-signaling downstream molecules or TLR5 itself. However, a clear and straight dose–response correlation was not seen.

The PAMP FLA is the main protein of bacterial flagellum, an appending organelle that commonly serves in locomotion and sensory functions for several Gram-positive and -negative bacteria [16,30]. The first-line innate immune response to the FLA is mediated by TLR5 [31], followed by intracellular downstream signaling. Upon NF-κB and MAP-kinase signaling pathways and the consecutive release of local and systemic immune-modulating cytokines like IL1β or TNFα, there is an interplay on innate and adaptive immune response detectable [32]. As the influences of LPS on cardiomyocytes and the heart are broadly known, there are just a few studies showing the influences of FLA explicitly on cardiac tissue in the literature, mainly using rat cardiomyocytes and whole heart extracts [10,33]. In addition to that, a non-infectious role of TLR5 has also been shown to play a role in cardiac dysfunction [34]. Going along with these observations, we could confirm several aspects in our hiPSC-CM-based results, e.g., elevated expression levels for NF-κB subunits or caspase-8 and partly increased expression and supernatant levels for the major inflammation modulator TNFα. However, immune-modulating IL6, its main receptor CD126 and chemotaxis cytokine chemokine ligand 8 (CXCL8=IL8) were not increased by FLA in hiPSC-CMs. Furthermore, besides TNFα, there were no more FLA-associated cytokine increases detectable in supernatants. Likewise, apoptosis/necrosis or ROS alterations as indicators for inflammation-induced cellular damage have not been seen, while it must be mentioned that further apoptosis/necrosis measurements or studies of distinct cell death types such as necroptosis have not been performed [35]. Additionally, further immune-response modeling aspects like the reciprocal behavior of pro- and anti-inflammatory cytokines such as IL10 could not be revealed [36,37]. However, in summary, we could not reveal a clear dose–response correlation or pro- or anti-inflammatory trends, as reported for other PAMPs in distinct tissues [13,18]. Compared to those observations, our results might lead to the assumption that FLA is not a potent inflammation inducer or modulator, at least with the focus on cytokine response, apoptosis/necrosis induction and ROS alterations [13,22]. Interestingly, this phenomenon had already been described for another PAMP. LTA, which influences contractility in rat cardiomyocytes and induces ROS alterations in cardiac tissue [18,20], is discussed as having failing inflammatory abilities from in vitro purification [38], probably with regard to the LTA origin [39,40]. Corresponding to these observations, our results could give rise to the idea of a weaker in vitro inflammatory response triggerability of FLA. On the other hand, the used values could have been too low compared to previous studies [14], reflecting more myocarditis-like circumstances in our results.

However, in addition to FLA’s abilities, the hiPSC-based modeling itself must likewise be discussed in this context. While there are several positive aspects in hIPSC-CMs-based in vitro studies, such as unlimited cell tissue availability, several cell attributes in this approach must be considered [21]. As in vivo immune response is first mediated through interactions of different cell types, and furthermore, the differentiated hiPSC-CMs cultures are lacking in absolute homogeneity, the results cannot be as clearly attested as those from other PAMP–cardiomyocytes interactions [41]. However, it is not clear whether a purified cardiomyocyte-only in vitro culture would more properly reflect the in vivo cell composition of the heart, particularly with respect to an immature heart tissue status [42,43]. It might even allow more accurate determination of FLA–cardiomyocyte interactions. This important aspect should be addressed by future studies. However, a lower maturation level and indicators for an early developmental stage status of hiPSC are known [44,45]. It is also known from human embryonic stem cells that they require prolonged maturation times for immune qualification after being already differentiated into distinct functional cell types, going along with lower TLR levels [46]. Furthermore, other professional APC such as dendritic cells even require PAMP exposure for the maturation process [47]. These considerations might lead to the idea that FLA is not a weaker innate immune response initiator, but the hiPSC-CMs themselves could be immature, at least regarding innate immune response abilities. As we have observed increased receptor levels, not only for TLR5 but also for the adhesion molecules CD54 and CD106 as members of a further major receptor group involved in inflammatory cardiac pathways [10,48], this might support this assumption. In terms of clinical considerations, TLR5 upregulation due to FLA exposure might already be relevant enough in influencing cardiac remodeling processes, as TLR5 seems to be involved in such. FLA-exposure-associated TLR5 upregulation could be able to sensitize the cardiac tissue for further downstream signaling pathways in this manner [49]. Furthermore, the revealed downregulation of the expression levels in several ion-channel genes could lead to additional clinical considerations, as potential arrhythmic effects due to PAMP–ion channel interactions have already been described [50]. Therefore, and with respect to the overviewing abstract nature of the study design, we could attest innate immune response induction from FLA, on the one hand, but our results did not provide more precise conclusions from the available results, on the other hand, which makes, in summary, more detailed studies necessary to enlighten FLA-associated pathways in cardiomyocytes.

## 5. Conclusions

FLA-induced innate immune response processes in cardiomyocytes might be detectable with an hiPSC-CM based in vitro model.

## 6. Study Limitations

Several limitations of our study must be mentioned. Firstly, the generated hiPSC-CM cell culture consists mainly, but not solely, of cardiomyocytes. Even though we performed studies with the measurement of troponin-only-positive cells, several experiments were performed with a mixed cell culture. However, the further exploration of the cell composition was not the aim of this study (compare discussion). Interestingly, some interleukin alterations were already detectable in the absence of FLA, which should be addressed in further studies. Further technical aspects like subliminal effects or effects above the threshold resulting from the chosen FLA dose or the use of only one chemical FLA compound and wearing-off phenomena must be mentioned. Likewise, the use of only one housekeeping gene in terms of PCR must be listed as a further limiting factor to be considered. Our FLA dose and incubation duration might be too low or too short (compare discussions). Even though we have performed several preliminary studies addressing these points, our results must be interpreted considering those important potential limitations. Finally, the lack of more precise techniques like Western blotting or whole-cell patch clamping have to be listed as limiting factors of data generation and interpretation.

## Data Availability

Derived data supporting the findings of this study are available from the corresponding author [G.Y.] on request.

## Supplementary Materials

The following supporting information can be downloaded at: https://www.mdpi.com/article/10.3390/ijms241813933/s1.
