# Supplementary material for: Flagellin-Induced Immune Response in Human-Induced Pluripotent Stem Cell-Derived Cardiomyocytes
Source: Int J Mol Sci. 2023 Sep 11;24(18):13933. doi: 10.3390/ijms241813933 (PMC10531389; doi:10.3390/ijms241813933)
Supplement: Supplementary file 1 [file ijms-24-13933-s001.zip › ijms-2562216-supplementary.pdf]

Article

Flagellin-Induced Immune Response in Human-Induced Pluripotent Stem Cell-Derived Cardiomyocytes

Goekhan Yuecel [1,2,*,†], Xiaobo Zhou [1,2], Linda Terkatz [1,3], Angela Wendel [1,2], Julius Reinhardt [1,2], Ibrahim El-Battrawy [4], Katherine Sattler [1,2], Lukas Cyganek [5], Jochen Utikal [6,7], Harald Langer [1,2], Ruediger Scharf [1,2], Daniel Duerschmied [1,2] and Ibrahim Akin [1,2]

1 Department of Cardiology, Angiology, Haemostaseology and Medical Intensive Care, University Medical Center Mannheim, Medical Faculty Mannheim, Heidelberg University, 68167 Mannheim, Germany
2 European Center for AngioScience (ECAS) and German Center for Cardiovascular Research (DZHK) Partner Site Heidelberg/Mannheim, 68167 Mannheim, Germany
3 Department of Pediatric Surgery and Pediatric Urology, Children's Hospital of Cologne, 50735 Cologne, Germany
4 Department of Cardiology and Angiology, Bergmannsheil University Hospitals, Ruhr University of Bochum, 44791 Bochum, Germany
5 DZHK~(German Center for Cardiovascular Research), Partner Site, 37075 Göttingen, Germany
6 Stem Cell Unit, Clinic for Cardiology and Pneumology, University Medical Center Göttingen, 37075 Göttingen, Germany
7 Skin Cancer Unit, German Cancer Research Center (DKFZ), 69120 Heidelberg, Germany
8 Department of Dermatology, Venereology and Allergology and DKFZ Hector Cancer Institute, University Medical Center Mannheim, University of Heidelberg, 68167 Mannheim, Germany
* Correspondence: goekhan.yuecel@medma.uni-heidelberg.de
† Current address: First Department of Medicine, University Medical Center Mannheim, Theodor-Kutzer-Ufer 1-3, 68167 Mannheim, Germany.

Supplementary Data

Supplementary Table 1

**Manufacturer/ Product name**	**Catalog/Product No**
**AdipoGen Life Sciences**	
Staurosporine	AG-CN2-0022
**AppliChem**	
EDTA solution pH 8.0 (0.5M)	A4892,0500
**BD Biosciences**	
BD Perm/Wash	554723
**Biological Industries**	
RPMI 1640 w/o L-Glutamine / D-Glucose	01-101-1A
**Bioron**	
SibirRoxHot Master Mix	119405
**Carl Roth**	
Roti®-Histofix 4 % (Formaldehyd)	P087.5
**Corning**	
Corning® Matrigel® Growth Factor Reduced Basement Membrane Matrix	354230
Centrifuge tubes	11376044
**Falcon**	
Culture Slides	354114
**Gibco™ / Thermo Fischer Scientific / Merck**	
Penicillin-Streptomycin (10.000 U/mL)	15140122
RPMI Medium 1640 (1x) + L-GlutaminTM Medium	11875085
RPMI Medium 1640 (1x) + GlutaMaxTM Medium	61870010
Sodium Pyruvate (100 mM)	11360039

B-27® Supplement (50X), serum free	17504044
HEPES (1 M)	15630080
2-Mercaptoethanol (50 mM)	31350010
DPBS, no calcium, no magnesium	14190144
DPBS, with calcium, with magnesium	14040091
RNaseOUT™ Recombinant Ribonuclease Inhibitor	10777019
dNTP Mix (10 mM)	18427013
MicroAmp™ Fast Optical96-well Collection Plate	4346907
Fetal Calf Serum	10270098
Sodium DL-lactate solution 50 %	72713
Total ROS Assay Kit	88-5930-74
**Miltenyi Biotec**	
StemMACS CHIR99021	130-103-926
Human FGF-2, premium grade	130-093-841
StemMACS Y27632	130-103-922
**Qiagen**	
RLT Lysis Buffer	160025243
RNeasy Mini Kit	74106
RNase-Free DNase Set	79254
Collection Tubes	BB18005
QIAshredder	79656
**RayBiotech**	
ELH-IL1β	P01584
ELH-IL6	P05231
ELH-TNFα	P01375

ELH-Troponin T	P13805
**R&D Systems**	
Recombinant Human/Mouse/Rat Activin A Protein	338-AC-010
Recombinant Human BMP-4 Protein	314-BP-010
**Roche**	
AMV Reverse Transcriptase with Buffer	10109118001
oligo-p(dt)$_{15,}$ Primer for cDNA Synthesis	10814270001
**Sigma-Aldrich**	
Flagellin from Salmonella typhimurium	SRP8029
Bovine Serum Albumin	10711454001
PCR dNTP Nucleotide Mix	11581295001
L-Ascorbic acid 2-phosphate sesquimagnesium salt hydrate	A8960-5G
Sodium DL-lactate	BCBQ6934V
Trypan Blue Solution	21K2376
0,5% Triton	X-100
**Stemcell Technologies**	
TeSR™-E8™	05990
IWP-4	72552
**Stemgent**	
ROCK Inhibitor (Y27632)	04-0012
Wnt Inhibitor IWP-4	04-0036
**Vector Laboratories**	
VECTASHIELD with DAPI	VEC-H-1200

Supplementary Table 1. List for each molecular biological material used in the studies sorted by manufacturer and affiliated product number. EDTA = Ethylenediaminetetraacetic acid, RPMI = Roswell Park Memorial Institute medium, HEPES = N-(2-Hydroxyethyl)piperazine-N′-(2-ethanesulfonic acid), HEPES = N-(2-Hydroxyethyl)piperazine-N′-(2-ethanesulfonic acid), DPBS = Dulbecco's phosphate buffered saline, dNTP = deoxyribose nucleotide triphosphate, ROS = Reactive Oxygen Species, FGF-2 = fibroblast growth factor beta, ELH = Human ELISA, IL = Interleukin, TNFα = Tumor necrosis factor α, BMP4 = Bone morphogenetic protein 4, AMV = Avian Myeloblastosis virus, DAPI = 4′,6-diamidino-2-phenylindole.

Supplementary Table 2

Abbreviation	Name	GeneID	Catalog No
ACTN2	Actinin alpha 2	88	PPH10284A
CACNA1C	Calcium voltage-gated channel subunit alpha1 C	775	QT00053480
Casp8	Caspase 8	841	PPH00359F-200
CCL2	C-C motif chemokine ligand 2	6347	PPH00192F-200
CTF1	Cardiotrophin 1	1489	PPH02069F-200<br>QT02318533
GAPDH	Glyceraldehyde-3-phosphate dehydrogenase	2597	PPH00150F-200<br>QT00079247
GJA1	Gap junction protein alpha 1	2697	PPH02781E-200
GJA5	Gap junction protein alpha 5	2702	PPH06113F-200
GJA7	Gap junction protein gamma 1	10052	PPH02456E-200
HCN2	Hyperpolarization activated cyclic nucleotide gated potassium and sodium channel 2	610	PPH06874B-200
HCN4	Hyperpolarization activated cyclic nucleotide gated potassium channel 4	10021	PPH14624C-200<br>QT00038108
IL1β	Interleukin 1 beta	3553	PPH00171C-200
IL6	Interleukin 6	3569	PPH00560C-200
IL8	C-X-C motif chemokine ligand 8	3576	PPH00568A-200
IL10	Interleukin 10	3586	PPH00572C-200
IL18	Interleukin 18	3606	PPH00580C-200

KCND3	Potassium voltage-gated channel subfamily D member 3	3752	PPH06923A-200
KCNJ3	Potassium inwardly rectifying channel subfamily J member 3	3760	PPH01412A-200
MAPK1	Mitogen-activated protein kinase 1	5594	PPH00715B-200
MAPK8	Mitogen-activated protein kinase 8	5599	PPH00720B-200
MAPK14	Mitogen-activated protein kinase 14	1432	PPH00750B-200
MYBPC3	Myosin binding protein C3	4607	PPH15269A
MyD88	MYD88 innate immune signal transduction adaptor	4615	PPH00911B-200
NANOG	Nanog homeobox	79923	PPH17032E
NCX1	Solute carrier family 8 member A1	6546	PPH12509B-200
NF-κB-1	Nuclear factor kappa B subunit 1	4790	PPH00204F-200
NF-κB-Rel-A	RELA proto-oncogene, NF-kB subunit	5970	PPH01812B-200
NKX2-5	NK2 homeobox 5	1482	PPH02462A
NLRP3	NLR family pyrin domain containing 3	114548	QT00029771
POU5F1	POU class 5 homeobox 1	5460	PPH02394E
SCN3B	Sodium voltage-gated channel beta subunit 3	55800	QT00002184
SCN5A	Sodium voltage-gated channel alpha subunit 5	6331	PPH01671F-200
SCN10A	Sodium voltage-gated channel alpha subunit 10	6336	PPH15064A-200
SOX2	SRY-box transcription factor 2	6657	PPH02471A

TLR5	Toll like receptor 5	7100	PPH01793F-200
TNFα	Tumor necrosis factor	7124	QT00029162
TNNI3	Troponin I3, cardiac type	7137	PPH02622G
TNNT2	Troponin T2, cardiac type	7139	PPH02619A

Supplementary Table 2. Abbreviation and full names of all human genes, which were measured in PCR studies, are listed in supplementary Table 2. GeneID: reference number from National Center for Biotechnology Information (NCBI) gene-database. Catalog No: RT2 qPCR Primer Assays and QuantiTect Primer Assays catalog number (Qiagen).

Supplementary Table 3

Manufacturer	Name	Catalog No
BD Biosciences	BD FACSCanto™ II	338960
Kendro Laboratory	HERAcell 240 Incubator	5536009
Leica Microsystems	DM3000 Microscope	090-136.001
Stratagene	Mx3000P Multiplex qPCR System	401405
Tecan	Inifinite M200 Microplate Reader	30016056

Supplementary Table 3. List of used laboratory apparatus and equipment.

Supplementary Table 4

Manufacturer, Name	Catalog No
**Biolegend**	
Anti CD106 (APC)	305810
Anti CD126 (PerCP/Cy5.5)	352812
**Biorbyt**	
Anti-Cardiac TroponinT (FITC)	orb187249
**BD Biosciences**	
Anti-Cardiac TroponinT (AF647)	565744
Anti-TLR5 (AF647)	564344
Anti-Annexin-V (FITC)/Propiumiodid kit	556547
Anti CD31 (AF647)	558094
Mouse Anti CD62P w/o conjugate	555524
Anti-Nf-κB p65 (AF488)	558421
**Novus Biologicals**	
Anti-Alpha Actinin 2 (AF647)	NBP3-08584AF647
**R&D Systems**	
Anti NLRP3 (AF647)	IC7578R
**Rockland Immunchemicals**	
Goat Anti-Mouse	ABIN2145562
Anti CD54 (FITC)	ABIN2144636
Mouse Anti CD130 w/o conjugate	ABIN1383683
**Supplementary Table 4.** List of used antibodies for FACS and immunohistochemistry sorted by manufacturer and affiliated product number. The conjugated fluorescence-compounds are listed in brackets. Unless not mentioned otherwise, all listed antibodies are targeting the corresponding human protein of the gene. Due to overview reasons, antibodies' hosting species are not listed. TLR	

= Toll Like Receptor, AF = Alexa Flour TM, CD = cluster of differentiation, PerCP/Cy5.5 = Peridinin chlorophyll protein-Cyanine5.5, FITC = Fluorescein isothiocyanate, Nf-κB p65 = nuclear factor kappa-light-chain-enhancer of activated B cells, p65 subunit, NLRP3 = NLR family pyrin domain containing 3.

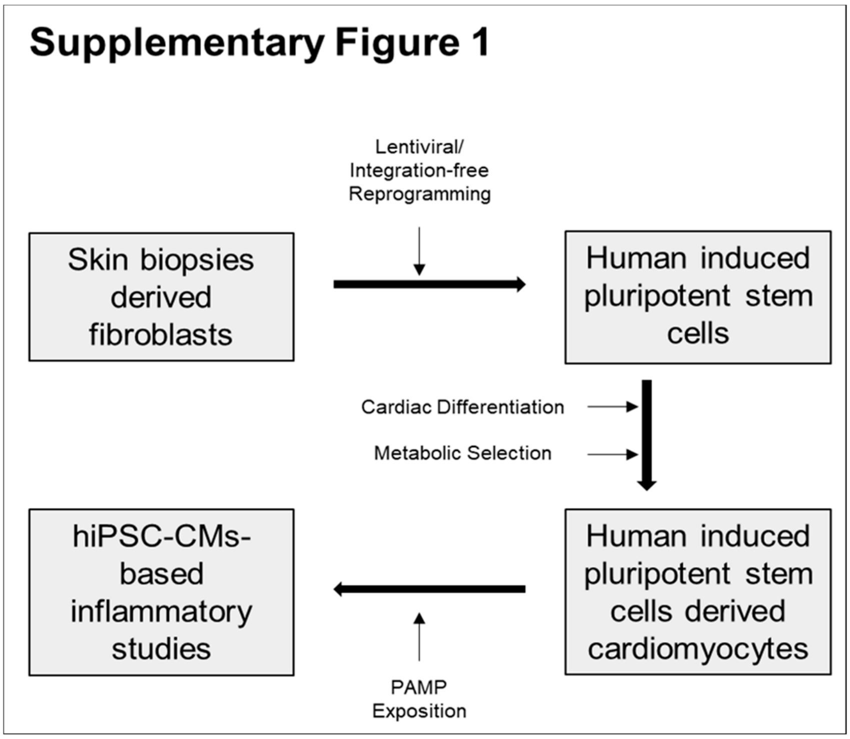

Supplementary Figure 1. Schematic overview and workflow for hIPSC-CMs-based inflammatory studies.

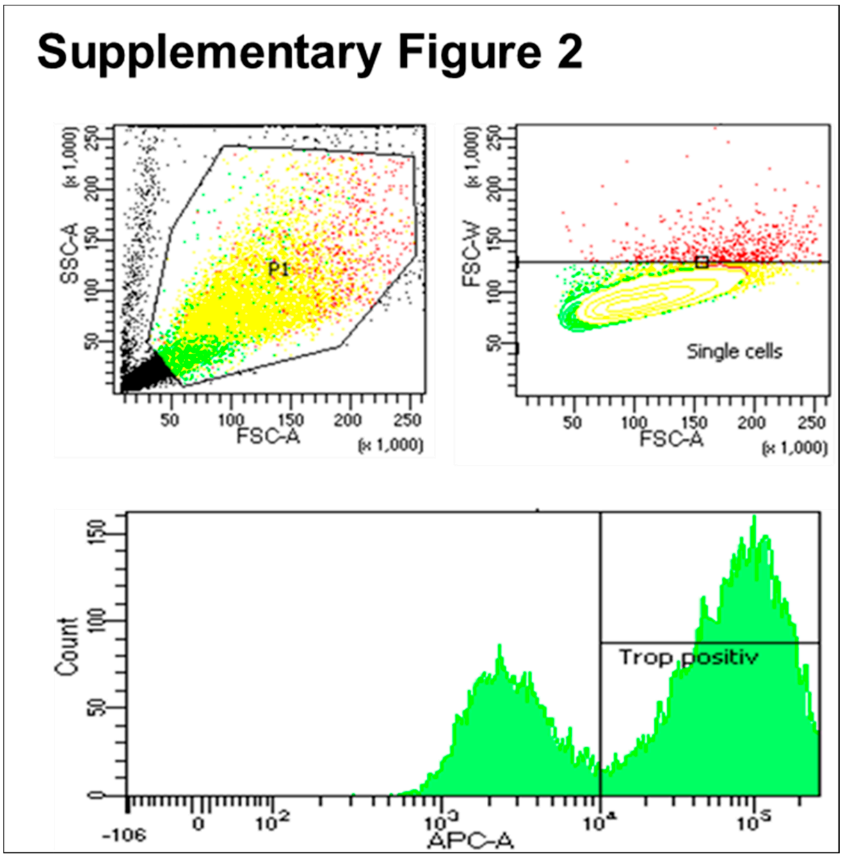

Supplementary Figure 2. Exemplary illustration for gating-order in FACS measurement of hiPSC-CMs with AF647-conjugated Anti-Troponin T (cardiac type) antibody during cardiac differentiation process on day 30. Forward scatter (FSC), sidewards scatter (SSC), signal width(-W) and area (-A) were used to define single cell events within measurement population P1, followed by definition of antibody-positive binding signal based on Mean Fluorescence Intensity (MFI).

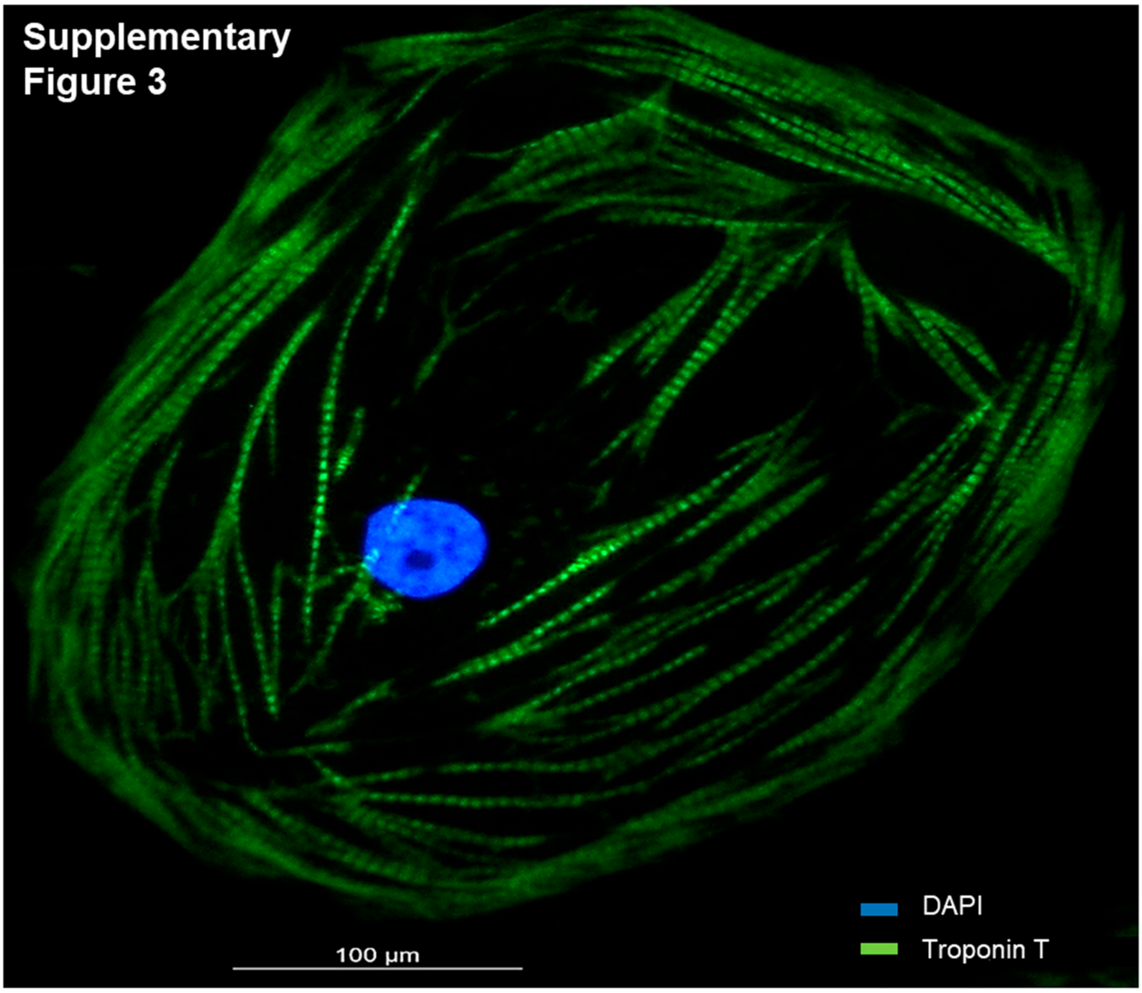

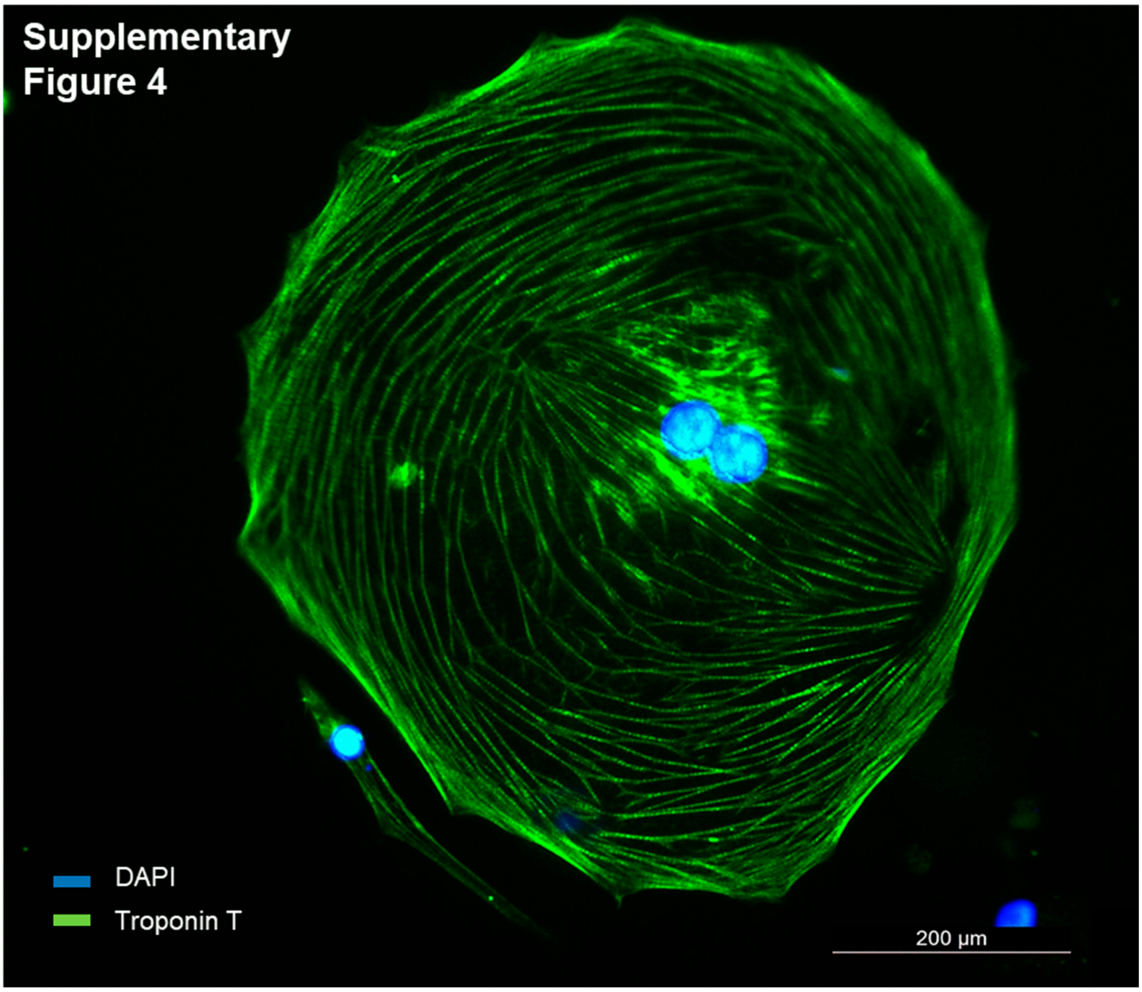
Supplementary
Figure 4
DAPI
Troponin T
200 µm

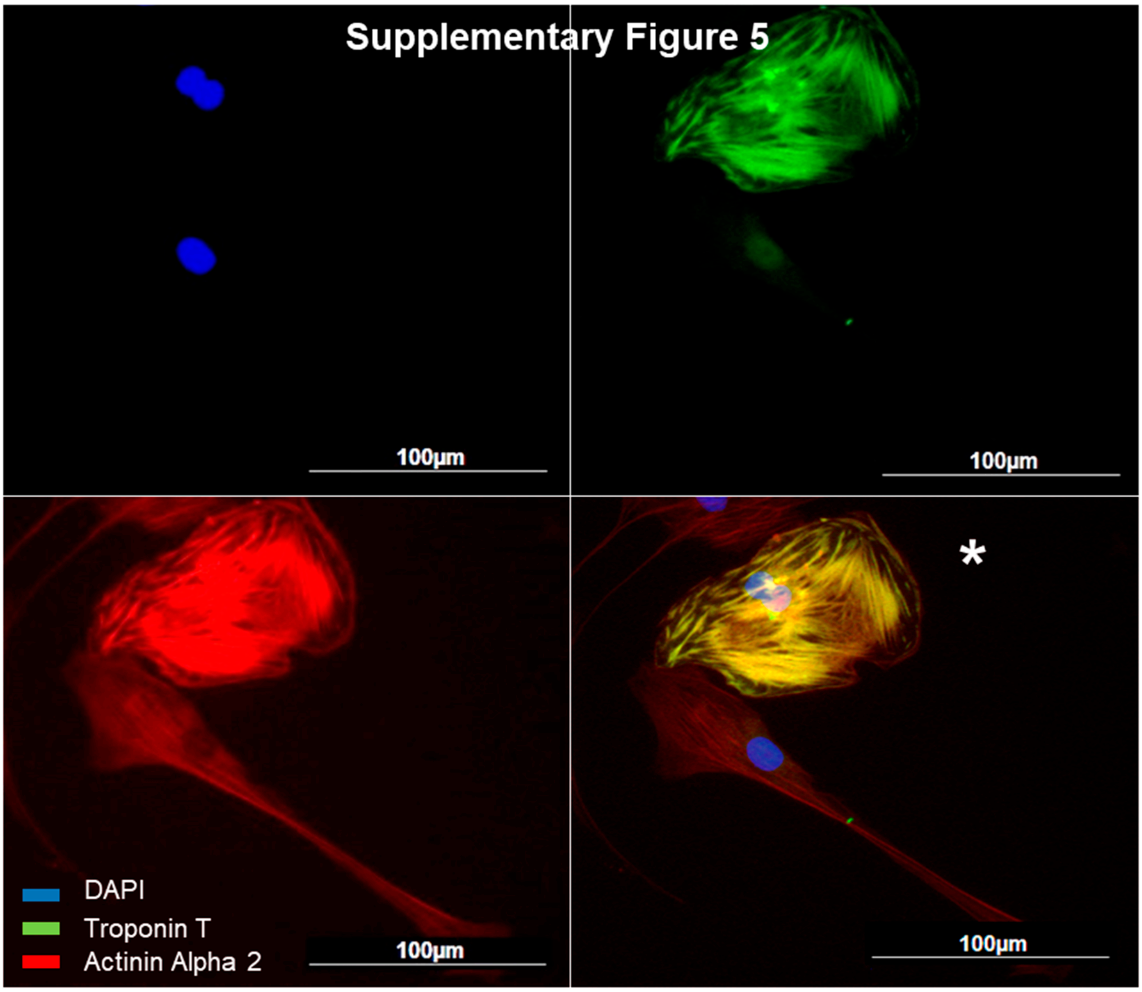

Supplementary Figure 3-5. Immunostaining of hiPSC-CMs at day 25 after begin of differentiation process. Blue: DAPI. Supplementary Figure 3,4: Staining for cardiac Troponin T (green, FITC Anti-TNNT2) illustrates typical striated morphology due to sarcomeres-formation (3, scale bars 100µm) and implied spindle apparatus due to cell division ability (4, scale bars 200µm). Supplementary Figure 5: Staining for cardiac Troponin T (green, FITC Anti-TNNT2) and Actinin Alpha 2 (red, AF647 Anti-ACTA2) implies in merge-illustration (*) the cell-cell proximity of troponin-positive and troponin-negative cells (scale bars 100µm).
